# JEV Infection Induces M-MDSC Differentiation Into CD3^+^ Macrophages in the Brain

**DOI:** 10.3389/fimmu.2022.838990

**Published:** 2022-04-21

**Authors:** Nan Zhang, Xiaochen Gao, Weijia Zhang, Junyao Xiong, Xiaojian Cao, Zhen F. Fu, Min Cui

**Affiliations:** ^1^ State Key Laboratory of Agricultural Microbiology, College of Veterinary Medicine, Huazhong Agricultural University, Wuhan, China; ^2^ Key Laboratory of Preventive Veterinary Medicine in Hubei Province, The Cooperative Innovation Center for Sustainable Pig Production, Wuhan, China; ^3^ Key Laboratory of Development of Veterinary Diagnostic Products, Ministry of Agriculture of the People’s Republic of China, Wuhan, China; ^4^ International Research Center for Animal Disease, Ministry of Science and Technology of the People’s Republic of China, Wuhan, China

**Keywords:** Japanese encephalitis virus, M-MDSCs, CD3 macrophages, IRF7, CCL2

## Abstract

Japanese encephalitis virus (JEV) is one of the most important members of the flavivirus family. It is a typical zoonotic pathogen that has caused substantial social and economic losses worldwide. The relation between JEV-induced immunosuppression and inflammatory responses has not been thoroughly investigated. In this study, cells infiltrating the brain tissue of JEV-infected mice were mainly identified as monocytic myeloid-derived suppressor cells (M-MDSCs), which subsequently differentiated into CD3^+^ macrophages. Co-culture with T cells showed that both splenic M-MDSCs and brain infiltrated M-MDSCs isolated from JEV-infected mice inhibited T cell proliferation through ARG1 and iNOS. The splenectomy model revealed that JEV-induced M-MDSCs were mainly derived from bone marrow and migrated to the spleen and central nervous system (CNS). The results of the transcriptome analysis and IRF7-deficient mice indicated that the ZBP1-IRF7 signaling pathway stimulated by JEV RNA played a central role in the induction of M-MDSCs. M-MDSCs migrated into the CNS through the chemokine CCL2/N-CCL2 derived from astrocytes and brain infiltrated M-MDSCs differentiated into CD3^+^ macrophages through a mechanism mediated by M-CSF, IL-6 and IFN-γ in the brain microenvironment. These findings provide evidence for the mechanism that JEV regulates the differentiation of M-MDSCs and thereby exacerbates pathogenicity, which represents a potential therapeutic target for Japanese encephalitis (JE).

## Introduction

Japanese encephalitis virus (JEV) belongs to the *Flavivirus* genus of the *Flaviviridae* family, identified as positive, single-stranded, enveloped RNA viruses, which is responsible for encephalitis-related morbidity and mortality worldwide. Members of the mosquito-transmitted viruses in this family cause encephalitis and hemorrhagic diseases, including JEV, West Nile virus (WNV), dengue virus (DENV), yellow fever virus (YFV), Zika virus (ZIKV), tick-borne encephalitis virus (TBEV), and St. Louis encephalitis virus (SLEV) (1). Among these viruses, JEV is the leading cause of viral encephalitis in the Asia-Pacific area, causing approximately 68,000 cases of Japanese encephalitis (JE) each year with the observed fatality rate of 30%. Almost 50% of JE survivors still suffer from permanent neuronal disorders, such as cognitive, motor, and behavioral impairments (2, 3). With climate change and globalization, JEV is still a significant threat to human and veterinary public health (4–6). However, to date, a specific drug treatment for JE is unavailable.

The virulence of JEV depends on its ability to disrupt and cross the blood-brain barrier (BBB) and to trigger inflammation in the central nervous system (CNS). After JEV crosses the BBB, various peripheral immune cells infiltrate the CNS (7–9), accompanied by the generation of numerous inflammatory factors and chemokines (10). Different studies have reported that CCR2^+^ T cells (7) and CD11b^+^Ly-6C^hi^ monocytes (8) are present in the mouse CNS during JEV infection. Similar CD11b^+^Ly-6C^+^ monocytes have also been identified as microglial precursors in WNV encephalitis (11), which is also an important member of the *Flaviviridae* family. Meanwhile, the CCR2 ligand CCL2 is produced by hematopoietic stem cell-derived leucocytes during JEV infection (8). However, JEV escape from immune surveillance and amplification in dendritic cells (DCs) and macrophage cells are the basis of what is described above. The interrelation and conversion between JEV-induced peripheral immunosuppression and the CNS inflammatory response remain ambiguous. An experimental inquiry into how JEV hijacks the immune response is helpful for the prevention and treatment of JE and other viral diseases. Myeloid-derived suppressor cells (MDSCs) are a widely accepted academic concept that was first reported in 2007 (12, 13) and refers to heterogeneous myeloid cells with the ability to suppress T cells. MDSC research initially focused on the cancer field, and these cells subsequently emerged as therapeutic targets for infectious diseases (14). A recent outbreak of COVID-19 has also been reported to be accompanied by dysregulated myeloid cell compartments and the expansion of myeloid-derived suppressor cells (15, 16). Based on their phenotypic and morphological features, MDSCs are mainly divided into two subsets, monocytic (M-MDSCs) and polymorphonuclear (PMN-MDSCs). Another subset that lacks macrophage and granulocyte markers, named early-stage MDSCs (e-MDSCs), also accumulates in several diseases (17). Similar to normal myeloid cells, MDSCs generally retain plasticity. Accumulating evidence strongly supports that MDSCs play an important role in viral infectious diseases. As shown in our previous study, MDSCs facilitate the progression of JEV infection (18). However, the pathway of JEV inducing MDSC production, and the association between MDSCs and JE pathology remains unclear.

Macrophages and T cells are generally considered to belong to different cell lineages. Meanwhile, it was reported that a novel macrophage subpopulation expressing CD3 molecule in humans and mice (19, 20) was discovered. These cells also express TCR and other molecules that are necessary for TCR signaling on lymphocytes. CD3 expression on macrophages is regulated by TNF and cholesterol import/export (20–22). Currently, limited reports on CD3^+^ macrophages are available. Previous studies suggest that CD3^+^ macrophages are involved in infectious, inflammatory and neoplastic diseases (23–25). In different studies, CD3^+^ macrophages and TCR^+^ macrophages have also been reported. As a new field of immunology, many questions remain unsolved regarding CD3^+^ macrophages.

In this study, we analyzed immune cells infiltrating the brains of JE mice using flow cytometry. JEV infection resulted in M-MDSC infiltration into the CNS, and the infiltrated M-MDSCs later differentiated into CD3^+^ macrophages. RNA-seq data and *in vitro* experiments indicated that the ZBP1-IRF7 signaling pathway plays an important role in JEV-induced M-MDSC generation. As transcription factor regulating interferon response, interferon regulatory factors (IRFs) catch researcher’s attention in the study of immune cell differentiation. Previous reports have proved that IRF4 and IRF8 can regulate MDSCs development and function (26, 27). Moreover, our results suggested that JEV RNA activated the ZBP1-IRF7 pathway. Furthermore, CD3^+^ macrophage differentiation was induced by M-CSF, IL-6 and IFN-γ. Moreover, the CCR2^+^ M-MDSCs responded to the ligand CCL2 mainly expressed on astrocytes after JEV infection with modified nitration. Based on these findings, we depict one pathway by which JEV regulates M-MDSC generation and differentiation due to its pathogenic capacity. All these studies are helpful to improve our knowledge and comprehension of the interaction between pathogens and the immune system.

## Materials and Methods

### Mice and Ethics Statement

All animal experiments were approved by the Research Ethics Committee, Huazhong Agricultural University, Hubei, China (HZAUMO-2019-060) and were performed in accordance with the Guidelines for the Care and Use of Laboratory Animals of the Research Ethics Committee, Huazhong Agricultural University, Hubei, China. WT C57BL/6 mice were obtained from Laboratory Animal Services Centre (Huazhong Agricultural University). C57BL/6-GFP mice were obtained from Shanghai Model Organisms Center (Shanghai, China). IRF7 KO mice were a kind gift from Prof. Ling Zhao (Huazhong Agricultural University).

### Viruses and Cell Lines

The JEV P3 and SA14-14-2 strains were previously preserved in our laboratory. The rAT full-length infectious JEV cDNA plasmid clone was a kind gift from Prof. Shengbo Cao (Huazhong Agricultural University). For viral proliferation, 1 × 10^3^ plaque-forming units (PFU) of JEV virus in 10 µL of Dulbecco’s modified Eagle’s medium (DMEM) were intracerebrally injected into the brain of suckling mice. The mice were sacrificed, and brains were collected from moribund mice. DMEM at a 10-fold volume of the brain weight was added, and the brains were homogenized on ice. The homogenate was centrifuged at 7,000 × g for 45 min, and the supernatant was aliquoted and stored at −80°C. The viral titer was determined by plaque formation assays with a baby hamster kidney fibroblast cell line (BHK-21), as previously described (28, 29). All cell lines used in this research were cultured in Dulbecco’s modified Eagle’s medium (DMEM) supplemented with 10% fetal bovine serum (FBS), 100 IU/mL penicillin, 100 mg/mL streptomycin, and 40 μM β-mercaptoethanol (2-ME) and incubated at 37°C in a 5% CO_2_ atmosphere.

### Reagents

OptiPrep, a 60% (w/v) solution of iodixanol, was obtained from Axis-Shield (Dundee, Scotland). The FAM-labeled A-NA RNA oligomer 5′-ACGCGCGCGCGUU UUCGCGCGCGCGU-3′ was synthesized by Tianjin Biolino Acid Technology (Tianjin, China). JetMessenger, an RNA transfection reagent, was purchased from Polyplus (Illkirch, FRANCE). NOHA was obtained from Sigma (Beijing, China). The compound 1400W was purchased from Apex Bio (Houston, Unite States). Polyvinyl alcohol (P139535) was obtained from Aladdin (Shanghai, China). The antibodies used for Western blotting and ELISAs are described in **Supplemental Table 1**.

### Bone Marrow Transplantation

Bone marrow cells were harvested from femurs and tibias of 6-week-old C57BL/6-GFP mice. Recipient WT C57BL/6 mice were γ-irradiated with 8 Gy and reconstituted with 2 × 10^7^ GFP^+^ bone marrow cells *via* the tail vein infection (30). Mice were maintained in sterile cages containing autoclaved food and water supplemented with antibiotics. After 8 weeks after reconstitution, mice were tested for chimerism. Mice which reconstitution efficiencies were >80% as determined by FACS analysis of GFP+ blood leukocytes were subjected to JEV infection.

### Isolation of Cells From the Brain Tissue

The method of cell isolation from brain was referring to previous reports (31, 32). Perfused brains were minced gently and collected in 10 mL of DMEM containing 5% FBS, 0.05% collagenase I, and 10 μg/mL DNase I. The resulting slurry was mixed at 37°C for 2 hours and passed through a 40 μm mesh sieve. Another 2 mL of OptiPrep™ (Axis-Shield) was added to the suspension, which was pelleted at 400 × g for 10 min. The cell pellet was resuspended in 10% Optiprep-DMEM and pelleted again by centrifugation at 400 × g for 10 min. The cell pellet was resuspended in 5 mL of DMEM, carefully layered onto 16% Optiprep-DMEM, and centrifuged at 500 × g for 25 min. Cells were collected from the interface, diluted with an equal volume of DMEM, washed once and resuspended in DMEM prior to subsequent experiments.

### Mouse Splenectomy

To determine the derivation of brain invaded cells, splenectomy was performed. C57BL/6 mice were anesthetized by isoflurane inhalation. The splenic artery and vein were exposed and ligated. Later, the spleen was removed, and hemostasis was confirmed. Finally, the incision was sutured and sterilized. Mice were administered 0.5% penicillin/streptomycin in the drinking water for 10 days.

### Flow Cytometry

Single-cell suspensions from the brain, spleen, bone marrow and culture system *in vitro* were preincubated with 0.3 μg of anti-CD16/32 antibody for 10 min at room temperature. The sample was subsequently stained with different combinations of mAbs conjugated with fluorochromes in PBS containing 1% BSA for 30 min at 4°C. Cells were washed twice with 0.2% BSA in PBS. Cells were characterized using FACSCalibur (BD Biosciences) and Cytoflex (Beckman-Coulter) flow cytometers and sorted using MoFlo XDP (Beckman Coulter). The antibodies used for flow cytometry are described in **Supplemental Table 2**.

### RNA-Seq and Analysis

All RNA-seq samples were sorted and stored in TRIpure reagent (Aidlab) at -80°C prior to RNA extraction. RNA extraction and transcriptome sequencing were performed by BGI Genomics. Subsequent data analysis was performed using the online system provided by Dr. Tom. All RNA-seq data have been uploaded to the NCBI SRA database. All accession numbers are described in **Supplemental Table 3**.

### Quantitative Real-Time PCR and Gene Expression Profiling

Total RNA was extracted with TRIpure reagent according to the manufacturer’s protocol. The cDNA templates were synthesized using a THERMOscript RT Kit (Aidlab). Real-time PCR was performed using Genious SYBR Green Fast qPCR Mix (ABclonal) on a QuantStudio 5 real-time PCR system (Applied Biosystems). The expression of target mRNAs was normalized to β-actin and RPL7L1. The primers used for real-time PCR are described in **Supplemental Table 4**.

### Statistical Analyses

All data were analyzed with GraphPad Prism v5.0 software (GraphPad Software, La Jolla, CA). Data are presented as the means ± SEM, and the significance of differences between groups was evaluated using a parametric ANOVA with a Tukey posttest or a *t* test. A *p* value < 0.05 was considered significant: *p < 0.05, **p < 0.01, *** *p *< 0.001, and ****p < 0.0001.

## Results

### JEV Infection Induces M-MDSC Infiltration and Subsequent Differentiation Into CD3^+^ Macrophages in the Brain

**Figure 1:**
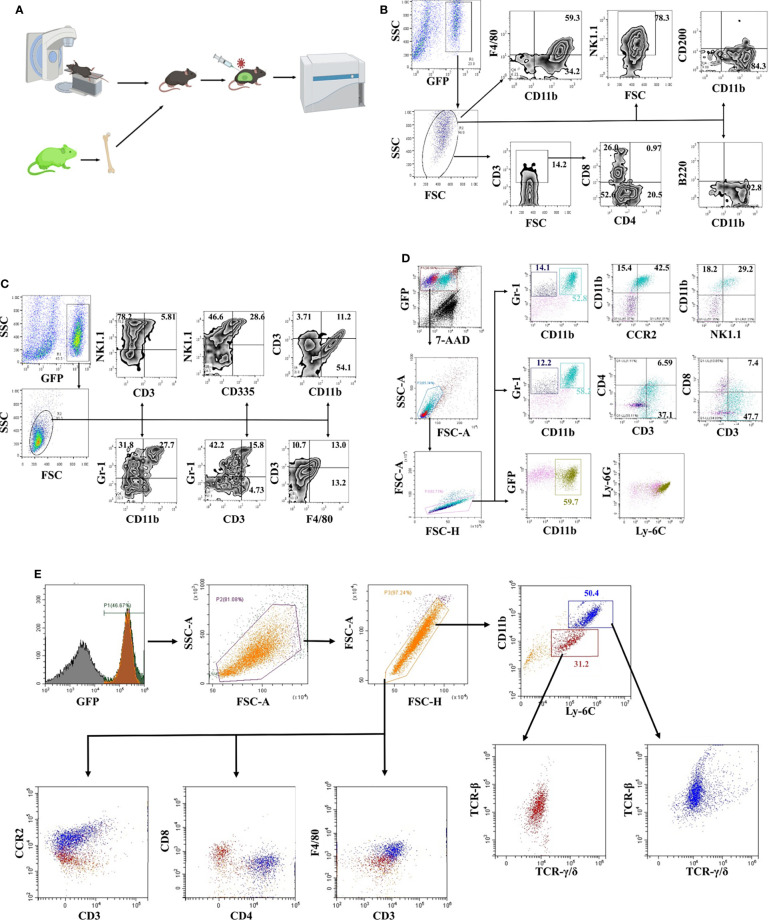
Phenotypic identification of infiltrating cells in the JE brain. **(A)** Schematic illustration of the bone marrow transplantation model. **(B)** Flow cytometry analysis of infiltrated cells in JE mouse brain tissue showing the presence of CD11b^+^, CD3^+^, and NK1.1^+^ cells. **(C)** Flow cytometry analysis of infiltrated cells in JE mouse brain tissue showing the presence of CD11b^+^Gr-1^+^, Gr-1^+^, and NK1.1^+^ cells. **(D)** Flow cytometry analysis of infiltrated cells in the JE mouse brain tissue showing the presence of CD3 and CCR2 on the surface of CD11b^+^Gr-1^+^ cells. **(E)** Flow cytometry analysis of infiltrated cells in the JE mouse brain tissue showing the presence of CD11b^+^Ly-6C^hi^F4/80^+^CCR2^+^CD3^+^TCR-β^+^TCR-γ/δ^+^ cells.

Chimeric mice were constructed with bone marrow from transgenic mice constitutively expressing enhanced green fluorescent protein (EGFP) to study the JEV-triggered inflammatory response in the CNS (**Figure 1A**). Eight weeks after transplantation, more than 80% PBMCs were GFP positive confirmed by flow cytometry detection (**Supplemental Figure 1A**). Bone marrow-transplanted mice were injected i.v. with the JEV P3 strain (5.0 × 10^4^ PFU). Once disease signs were shown (clinical score = 1 or 2), the mouse was sacrificed, and immune cells were isolated from brain tissues and observed under fluorescence microscopy (**Supplemental Figures 1B, C**). The inflammatory cells that infiltrated the brains of mice with JE were mainly CD11b^+^ cells (93%) and NK1.1^+^ cells (78%), along with a small percentage of CD3^+^ (14%) cells. Meanwhile, most CD3^+^ cells were CD4^-^CD8^-^ (**Figure 1B**). Furthermore, different combinations of cell surface markers were tested. The infiltrated inflammatory cells were mainly CD11b^+^Gr-1^+^ (28%), Gr-1^+^ (32%), and NK1.1^+^ (78%) cells (**Figure 1C**). Moreover, the CD335 expression level on NK1.1^+^ cells was only approximately 38% (**Figure 1C**). Based on these results, the immune cells infiltrating the brains of mice with JE expressed different cell lineage markers.

Hence, a more detailed phenotype identification was performed. The results from flow cytometry confirmed the presence of CD11b^+^Gr-1^+^ cells, which were considered as MDSC-like cells (13), in the brains of JE mice (**Figures 1C, D**). More interestingly, the majority of CD11b^+^Gr-1^+^ cells were CD3- and CCR2-positive. Gr-1 is comprised of Ly-6G and Ly-6C, representing the granulocytic and monocytic lineages, respectively.

Further analysis defined that the infiltrated cells were Ly-6C^+^ and Ly-6G^-^ (**Figure 1D**), consistent with other reports (8).

Afterwards, it was confirmed with flow cytometry analysis that CD11b^+^Ly-6C^+^ cells were the major cell population infiltrating the brains of JE mice. The infiltrated CD11b^+^Ly-6C^+^ cells were divided into two populations: approximately 30% of Ly-6C^+^ monocytes and 50% of Ly-6C^hi^ M-MDSCs (**Figure 1E**). Previous reports suggested that MDSCs did not express lineage-specific markers, but MDSCs can differentiate into DCs, macrophages, tumor-associated macrophages (TAMs), and granulocytes (33, 34). These M-MDSC-like cells isolated from JE mouse brains expressed F4/80 and CCR2 and the T cell lineage molecules CD3, TCR-β and TCR-γ/δ at different levels (**Figure 1E**). The CD3^+^F4/80^+^TCR^+^ phenotype was closer to CD3^+^ macrophages or TCR^+^ macrophages identified in recent years. Thus, JEV infection results in M-MDSC-like cell infiltration into the CNS, which subsequently differentiate into CD3^+^ macrophages.

### JEV Infection Induces M-MDSC Generation

**Figure 2:**
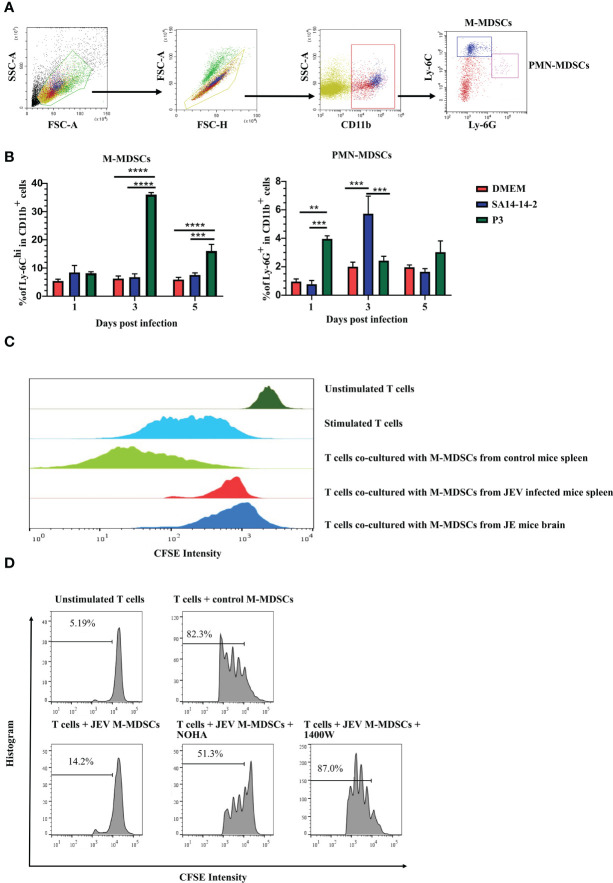
JEV infection expands M-MDSC generation. **(A)** Flow cytometry gating strategy for MDSCs. **(B)** Bar graphs show the M-MDSC and PMN-MDSC ratios in the spleen at 1, 3, and 5 days post-infection (n = 4). **(C)** M-MDSCs were sorted from the spleens of mock- and JEV-infected mice, and the brains of JE mice, then mixed with CFSE-labeled CD8^+^ T cells (pre-stimulated with 2.5 μg/mL ConA) at a ratio of 1:1. The inhibition of T cell proliferation was assessed after 3 days incubation at 37°C. The proliferation of CD8^+^ T cells was analyzed using flow cytometry. **(D)** Proliferation of CD8^+^ T cells in the presence of M-MDSCs from the spleen and NOHA or 1400W. Representative results from repeated experiments are shown. **p < 0.01, ***p < 0.001, and ****p < 0.0001.

M-MDSCs in peripheral tissues were measured after JEV infection to explore how JEV induced M-MDSC generation. Mice were infected with 1.0 × 10^5^ PFU of JEV P3. DMEM or the vaccine strain SA14-14-2 was injected at the same volume or dosage as the blank or negative control, respectively. The proportions of M-MDSCs and PMN-MDSCs in the spleen were measured using flow cytometry at the early stage of infection (**Figure 2A**). JEV P3 infection increased the M-MDSC proportion in the spleen on days 3-5 after infection, and the SA14-14-2 strain had no effect on M-MDSC generation (**Figure 2B**). In parallel, the P3 and SA14-14-2 strains respectively and transiently increased the PMN-MDSC proportion in the spleen on days 1 and 3 after infection, and then, PMN-MDSCs regressed quickly to normal levels (**Figure 2B**). Combined with the observation of M-MDSCs depletion (18), it suggests that M-MDSCs might contribute to the immune escape and pathogenic capacity of virulent P3 strains, and PMN-MDSC generation tends to be a systemic response to exogenous stimulation during JEV infection. Our previous study also confirmed that all-trans retinoic acid (ATRA) administration-mediated MDSC depletion increased the survival rate of mice after JEV infection (18).

To determine whether M-MDSC-like cells are capable of suppressing the T cell response, splenic CD8^+^ T cells (**Supplemental Figure 2A**) were activated and co-cultured with CD11b^+^Ly-6C^hi^ cells sorted from the spleen (**Supplemental Figure 2B**) at 4 days after JEV infection or JE brain tissue (**Supplemental Figure 2C**). T cell proliferation assays revealed that CD11b^+^Ly-6C^+^ cells isolated from the spleen and brain tissue of JEV-infected mice exerted a significant effect on suppressing T cell proliferation, while CD11b^+^Ly-6C^+^ cells from the spleen of mock-infected mice did not show the same capacity (**Figure 2C**). Thus, the CNS-infiltrated CD11b^+^Ly-6C^+^ cells from JEV-infected mice were functional MDSCs. The published literature suggests that the immunosuppressive activity of MDSCs is mainly associated with the production of Arg-1 and iNOS (35). Then, ARG1 inhibitor (NOHA) and iNOS inhibitor (1400W) were added to the co-culture system. Upon ARG1 or iNOS inhibition, the suppressive effects of JEV-induced M-MDSCs were dramatically decreased (**Figure 2D**).

A mouse splenectomy model was applied to explore the derivation of MDSCs in JEV-infected mice. Both splenectomized mice and normal mice were injected with 5.0 × 10^4^ PFU of JEV P3 or an equal volume of DMEM *via* the tail vein. The proportions of MDSCs in the blood, spleen, and bone marrow was evaluated on day 5 after the infection. Splenectomized mice showed significantly higher mortality rates than control mice, with a slightly prolonged disease course (**Supplemental Figure 3A**). After analysis, splenectomy surgery slightly increased the MDSC proportion in bone marrow without statistically significant, yet JEV infection also slightly increased the MDSC proportion in bone marrow without statistically significant. The proportion of MDSCs in the blood was increased slightly following splenectomy surgery and significantly increased following JEV infection (**Supplemental Figure 3B**). Thus, JEV-induced MDSCs are primarily derived from bone marrow.

### JEV Induces M-MDSC Generation Through the IRF7 Signaling Pathway

**Figure 3:**
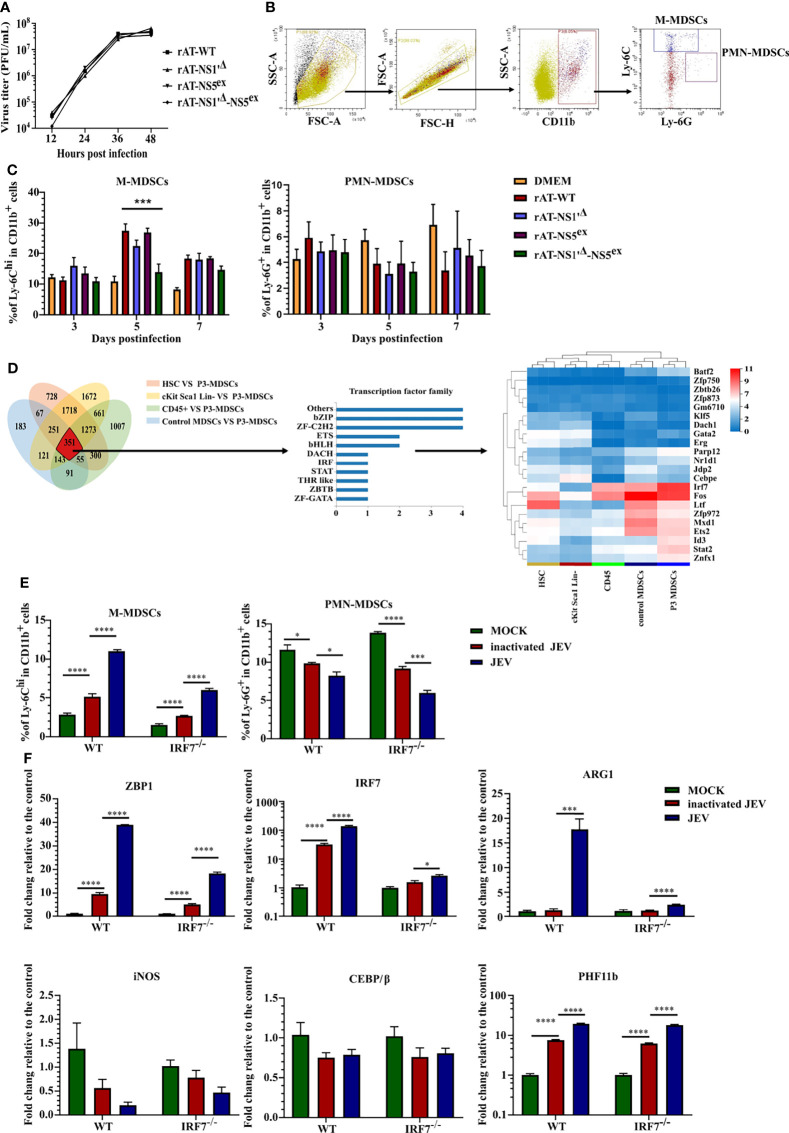
JEV induces M-MDSC generation through the IRF7 signaling pathway. **(A)** Replication kinetics of different rAT viruses in BHK-21 cells. **(B)** Flow cytometry gating strategy for MDSCs. **(C)** Bar graphs show the M-MDSC and PMN-MDSC ratios in the spleen after infection with different rAT viruses at 3, 5, and 7 days post-infection (n = 4). **(D)** Venn diagram and cluster heat map showing differentially expressed transcription factors among samples. **(E, F)** WT and IRF7 KO bone marrow cells were infected with inactivated or live JEV (MOI = 1). **(E)** The M-MDSC and PMN-MDSC ratios were determined using flow cytometry. **(F)** ZBP1, IRF7, ARG1, iNOS, CEBE/β, and PHF11b mRNA expression were determined using real-time PCR at 72 h post-infection (n = 4). Representative results of repeated experiments are shown. *p < 0.05, ***p < 0.001, and ****p < 0.0001.

All the results described above suggested that M-MDSCs play a pathogenic role in JEV infection. The underlying mechanism by which JEV induces M-MDSCs remains unknown. According to previous research, the JEV nonstructural proteins NS1' and NS5 are closely related to viral immune evasion and pathogenicity (36–38). Hence, reverse genetics and RNA-seq approaches were utilized to explore the mechanism of M-MDSC induction. The full-length JEV infectious cDNA clone rAT was used as the wild-type (WT) strain (39). The rAT-NS1'^Δ^ clone was constructed by introducing the NS2A-G66A mutation to prevent NS1' production (36). The rAT-NS5^ex^ clone was constructed by exchanging the NS5 gene from the AT31 strain to NS5 from the vaccine strain SA14-14-2. In addition, the clone combining NS5^ex^ and NS1'^Δ^ was also constructed and referred to as rAT-NS1'^Δ^-NS5^ex^. PCR (**Supplemental Figure 4A**) and enzyme digestion (**Supplemental Figure 4B**) confirmed the successful production of all constructs. All plasmids were transfected into BHK-21 cells with the T7 polymerase eukaryotic expression plasmid, and virus-containing supernatants were harvested 72 hours after transfection. The PrM protein of JEV was measured upon reinfection of BHK-21 cells (**Supplemental Figure 4C**). All rescued viruses were amplified in suckling mice and titered (40). NS2A and NS5 segment sequencing data from RT-PCR samples confirmed that all viruses were rescued successfully (**Supplemental Figure 4D**). Viral growth curves in BHK-21 cells indicated that there were no significant differences in replication among different viruses (**Figure 3A**).

Afterwards, mice were infected with rescued viruses *via* i.v. injection of 5.0 × 10^5^ PFU of the viruses. The proportions of M-MDSCs and PMN-MDSCs in the spleen were measured using flow cytometry (**Figure 3B**). No rAT strain induced PMN-MDSC generation. Meanwhile, the rAT-WT strain markedly induced the generation of M-MDSCs. Compared with the rAT-WT strain, the NS1'^Δ^ mutation slightly but not significantly attenuated M-MDSC induction, but the NS1'^Δ^-NS5^ex^ double mutation significantly weakened M-MDSC induction (**Figure 3C**). Based on these results, the NS1' and NS5 proteins cooperate to synergistically induce M-MDSC generation during JEV infection.

RNA-seq assays were performed on M-MDSCs sorted from the spleens of 1.0× 10^5^ PFU JEV P3-infected mice (P3 MDSCs), CD45^+^ cells (CD45) and M-MDSCs (control MDSCs) from the spleens of mock-infected mice, bone marrow cells (HSCs), and c-Kit^+^Sca-1^+^Lin^-^ cells (cKit Sca1 Lin-) from the bone marrow of mock-infected mice to further investigate the mechanism underlying M-MDSC induction by JEV, as shown in **Figure 3**.

The threshold was set to log_2_ FC≥ 1.0 and a Q value≤ 0.05. The Venn diagram showed 351 differentially expressed genes in P3 MDSCs compared with the other groups, among which 22 are transcription factors. A clustering analysis of the 22 transcription factor genes indicated that IRF7 was significantly upregulated in P3 MDSCs (**Figure 3D**). By analyzing the expression of the IRF family and interferon genes, only IRF7 was significantly upregulated in P3 MDSCs (**Supplemental Figure 5A**), and no discernible differences in the expression of interferon genes were observed among the different groups (**Supplemental Figure 5B**). The GSEA illustrated that IRF7 was included in the cytosolic DNA-sensing pathway (KEGG map04623), which was upregulated in P3 MDSCs (**Supplemental Figures 5C, D**).

Bone marrow cells from WT or IRF7 KO mice were infected with JEV P3 at an MOI of 1 *ex vivo* and continuously cultured for 3 days to study the role of IRF7 in MDSC induction; inactivated JEV-treated cells were used as a control. MDSC expansion and gene expression were assessed using flow cytometry (**Figure 3E**) and real-time PCR (**Figure 3F**), respectively. Ex vivo infection of bone marrow cells effectively expanded M-MDSCs. Bone marrow cells from IRF7^-/-^ mice still expanded into M-MDSCs, yet the ratio of M-MDSCs was lower than that of WT cells, especially after JEV infection. On the other hand, JEV infection slightly decreased the PMN-MDSC ratio in both types of bone marrow cells (**Figure 3E**). This difference may be attributed to an impairment in the bone marrow cell differentiation or proliferation of PMN-MDSCs due to M-MDSC generation.

Real-time PCR indicated that JEV infection significantly upregulated the expression of ZBP1 (DAI; DLM1), IRF7, PHF11b, and ARG1. Compared with wild-type bone marrow cells, IRF7 deficiency decreased the expression of ZBP1 and ARG1 but did not alter PHF11b expression. In addition, the CEBP/β gene, which has been reported to play an important role in MDSC generation (41–43), was not upregulated in JEV-induced MDSCs. Moreover, JEV infection downregulated iNOS expression, which was probably caused by antagonism and competition between ARG1 and the iNOS pathway (**Figure 3F**).

Thus, lentivirus vectors were constructed to achieve ZBP1 overexpression and different isoforms of IRF7. Overexpression of both ZBP1 and IRF7 induced M-MDSC production, but inhibited PMN-MDSC generation. However, among the 4 IRF isoforms, only IRF7-V1 induced significant ARG1 and iNOS upregulation (**Supplemental Figures 6A–C**). These results suggest that the ZBP1-IRF7 signaling pathway plays an important role in M-MDSC generation during JEV infection.

### JEV RNA Stimulates ZBP1 and IRF7 Expression

**Figure 4:**
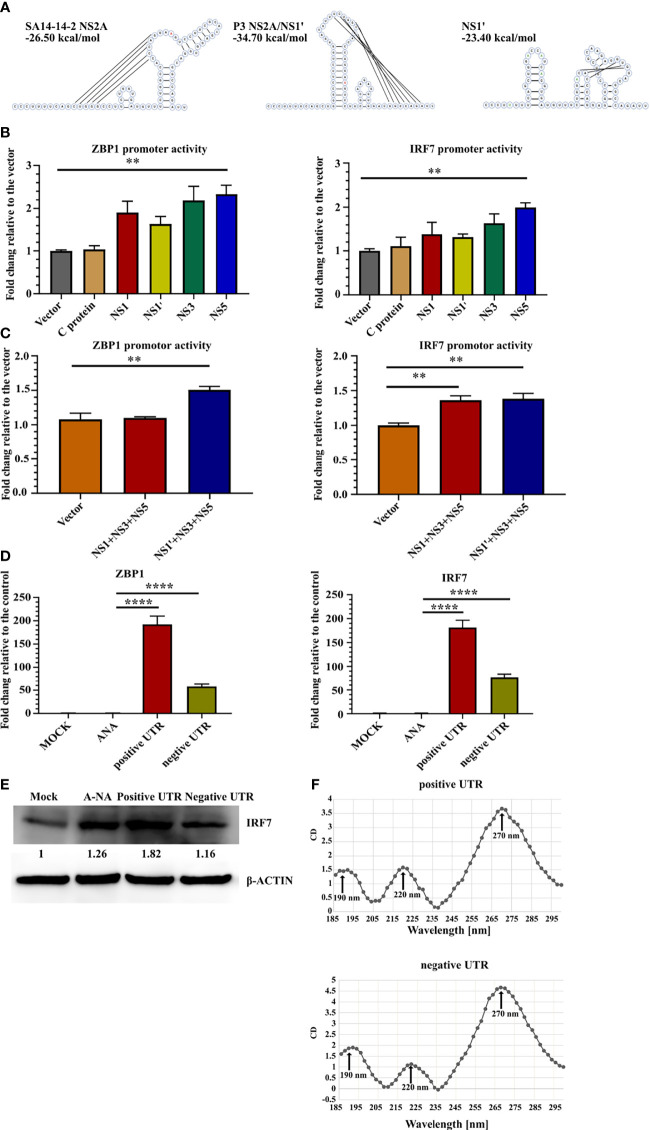
JEV RNA stimulates ZBP1 and IRF7 expression. **(A)** Predicted RNA structures of SA14-14-2 NS2A and pseudoknot structure P3 NS2A/NS1' and redesigned NS1' without the pseudoknot structure. Inserted and mutated bases are labeled in green and the 66th nucleotide of NS2A is labeled in red. **(B, C)** Dual-luciferase reporter system detection of ZBP1 and IRF7 promoter activity. **(B)** Bar graphs show the promoter activity of individual JEV proteins (n = 4). **(C)** Bar graphs show JEV transcription complex promoter activity (n = 4). **(D)** Real-time PCR detection of ZBP1 and IRF7 expression in C8-D1A cells after the transfection with different RNA sequences at 20 hours after transfection (n = 4). **(E)** Western blot showing IRF7 expression in C8-D1A cells at 20 hours after the transfection with different RNA sequences. **(F)** Circular dichroism spectra of JEV 3'-UTR RNA from 185 nm to 300 nm. Representative results from repeated experiments are shown. **p < 0.01, and ****p < 0.0001.

Next, the mechanism was explored to determine which viral factors induced ZBP1 and IRF7 upregulation. The transcriptional activity of NS1' and NS5 was studied (**Figures 3A–C**). Eukaryotic expression plasmids containing the genes of JEV C, NS1, NS1', NS3 or NS5 were constructed separately. Because NS1' production depends on the RNA pseudoknot secondary structure at the NS2A segment (44), the NS1' DNA sequence was designed to disrupt the pseudoknot, avoiding the structural ribosomal frameshift. Redesigned NS1 and NS1' sequences were analyzed using pKiss-BiBiServ2 (45) (**Figure 4A**).

The promoter regions of ZBP1 (-1201 to +135) and IRF7 (-1812 to +810) were cloned into the pGL3-basic vector and co-transfected with the protein expression plasmid into 293FT cells. pRL-TK was transfected as an internal control. A luciferase reporter assay showed that NS5 expression increased ZBP1 and IRF7 promoter activity, yet only an approximately 2-fold increase was observed (**Figure 4B**). NS1' is involved in the formation of JEV replication complexes (46). Thus, the JEV replication complex composed of NS1/NS1' NS3-NS5 may induce the transcription of ZBP1 and IRF7. Co-transfection of three plasmids, NS1', NS3 and NS5, slightly upregulated ZBP1 and IRF7 promoter activity (**Figure 4C**). Based on these results, JEV proteins are not the major inducers of ZBP1 and IRF7 expression.

The RNA-seq results revealed that many genes in the cytosolic DNA-sensing pathway were upregulated in P3 MDSCs (**Supplemental Figures 5C, D**). ZBP1 is one of these genes and contains two Zα domains that selectively bind to left-handed Z-form RNA. Previous studies reported the existence of subgenomic flavivirus RNA (sfRNA) during JEV infection, which is derived from the 3′-UTR of gRNA with a relatively complex structure and has been proven to contribute to viral evasion (47, 48). Therefore, we hypothesized that JEV RNA activates the transcription of ZBP1 and IRF7. Therefore, positive- and negative-strand JEV 3′-UTR fused partial NS5 C-terminal sequences were transcribed with a T7 transcription kit *in vitro* and transfected into C8-D1A cells. Meanwhile, mock-transfected and A-RNA (49)-transfected cells were used as controls. Real-time PCR detection found that compared with the control groups, 3′-UTR-transfected cells dramatically upregulated the expression of ZBP1 and IRF7 (**Figure 4D**). Western blotting also confirmed that positive 3′-UTR transfection significantly increased levels of the IRF7 protein (**Figure 4E**). Circular dichroism (CD) spectroscopy was used to examine the 3′-UTR structure. CD detection revealed that both 3′-UTR RNAs had one intensely positive CD band at 270 nm and two less intense positive CD bands at 190 nm and 220 nm (**Figure 4F**). However, this characteristic curve does not fit with the reported CD curve of oligonucleotides. This discrepancy was potentially caused by the more complex or mixed secondary structure of the JEV 3′-UTR RNA.

Taken together, these results indicated that JEV RNA induced M-MDSC generation through the IRF7 pathway, which contributed to viral immune escape.

### The JE Brain Microenvironment Causes CD3^+^ Macrophage Differentiation

**Figure 5:**
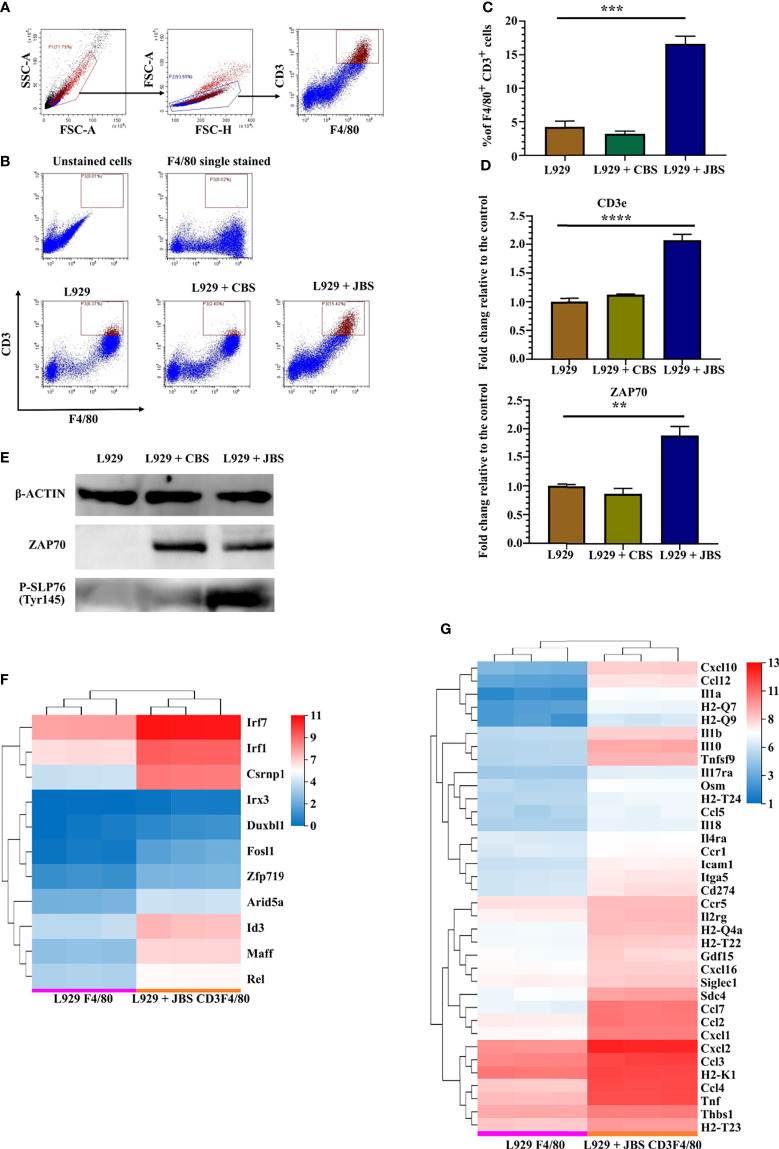
JE mouse brain homogenate supernatant induces CD3^+^ macrophage differentiation. **(A)** Flow cytometry gating strategy for CD3^+^ macrophages. **(B)** Representative scatter plots and **(C)** bar chart showing that JBS significantly induced CD3^+^ macrophage differentiation in an *ex vivo* induction system (n = 4). **(D)** Real-time PCR detection of CD3e and ZAP70 expression (n = 4) and **(E)** Western blots showing ZAP70 and P-SLP76 expression in bone marrow cells 4 days after JBS stimulation. Cluster heat map of differentially expressed **(F)** transcription factors and **(G)** cytokines between F4/80^+^ and CD3^+^F4/80^+^ cells. Representative results of repeated experiments are shown. **p < 0.01, ***p < 0.001, and ****p < 0.0001.

As infiltrated cells isolated from JE mouse brains have a phenotype similar to CD3^+^ macrophages (**Figure 1E**), an *in vitro* model was established to induce CD3^+^ macrophage differentiation with a JE mouse brain homogenate supernatant (JBS). Bone marrow cells were cultured in RPMI-1640 conditioned medium supplemented with 10% FBS and 2% L929 culture supernatants, and 3% JBS was added as a stimulator. The brain homogenate supernatant collected from uninfected mice (CBS) was used as a control. CD3^+^ macrophage differentiation was assessed using flow cytometry 4 days later. JBS stimulation significantly upregulated CD3 expression in F4/80^+^ macrophages (**Figures 5A–C**). Real-time PCR revealed CD3 and ZAP70 upregulation in the culture stimulated with L929 plus JBS (**Figure 5D**). Western blotting confirmed that both CBS and JBS stimulation increased levels of the ZAP70 protein, but only JBS stimulated SLP-76 phosphorylation (**Figure 5E**). These macrophages were labeled with CFSE and continuously stimulated with ConA for 48 hours. Then, the cell proliferation assay indicated a substantial increase in the proliferation of JBS-induced macrophages and a stronger response to ConA than untreated cells and CBS-treated macrophages (**Supplemental Figures 7A, B**). The fluorescence microsphere phagocytosis experiment indicated that JBS-induced CD3^+^ macrophages had a higher phagocytic ability (**Supplemental Figure 7C**).

RNA-seq was performed on sorted F4/80^+^ cells (L929 F4/80) and CD3^+^F4/80^+^ cells (L929 + JBS CD3F4/80) induced with L929 medium or L929 plus JBS medium to further study the mechanism of CD3^+^ macrophage generation during JEV infection. The threshold was set to log_2_ FC≥ 1.5 and a Q value≤ 0.05. Eleven transcription factor genes were upregulated in CD3^+^F4/80^+^ cells compared to F4/80^+^ cells (**Figure 5F**), among which IRF7, IRF1, and CSRNP1 were the most significantly upregulated genes. Moreover, GSEA revealed that the cytosolic DNA-sensing pathway (KEGG map04623) was upregulated in CD3^+^F4/80^+^ cells (**Supplemental Figures 8A, B**), similar to M-MDSCs (**Supplemental Figures 5C, D**). Meanwhile, various proinflammatory cytokines and chemokines were also upregulated in CD3^+^F4/80^+^ cells, including IL1α and IL1β, and some members of the TNF family, CCL family, and CXCL family (**Figure 5G**).

These results suggested that JEV infection and interferon molecules induce CD3^+^ macrophage generation, which in turn might contribute to neuroinflammation during JEV infection.

### M-CSF, IL-2, IL-6, and IFN-γ Induce CD3^+^ Macrophage Differentiation

**Figure 6:**
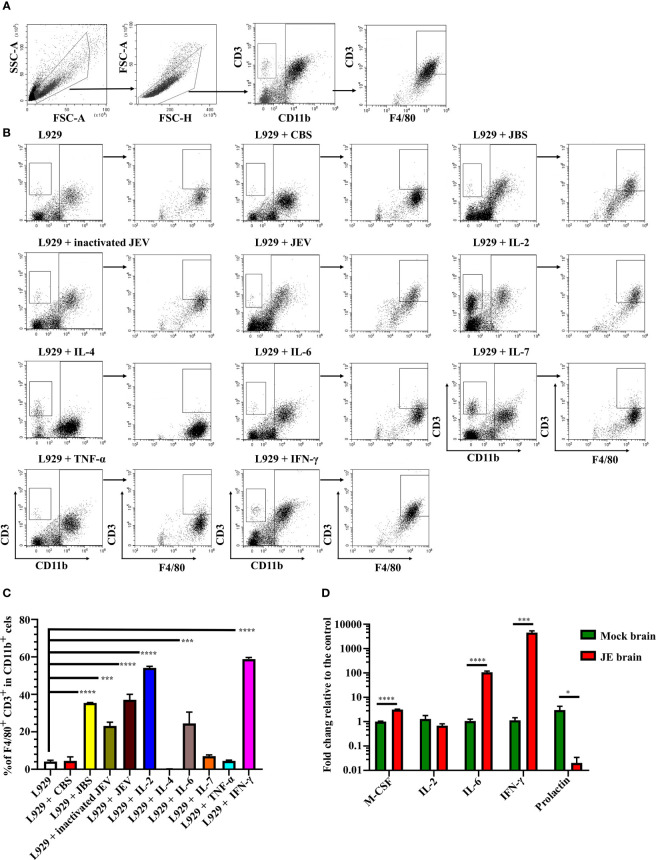
JEV infection upregulates M-CSF, IL-6, and IFN-γ to induce CD3^+^ macrophage differentiation. **(A)** Flow cytometry gating strategy for CD3^+^ macrophages. **(B)** Representative scatter plots and **(C)** bar chart show that L929 conditioned medium containing IL-2, IL-6, and IFN-γ significantly induced CD3^+^ macrophage differentiation in an ex vivo induction system at 4 days after stimulation (n = 4). **(D)** Expression of the M-CSF, IL-2, IL-6, IFN-γ, and prolactin mRNAs was determined using real-time PCR 4 days after infection (n = 4). Representative results of repeated experiments are shown. *p < 0.05, ***p < 0.001, and ****p < 0.0001.

We next verified whether JEV infection or interferon induced CD3^+^ macrophage differentiation. Bone marrow cells were cultured in the same conditioned medium as described above, and then 1 MOI of live JEV, inactivated JEV or 20 ng/mL different cytokines was added to the system. CD3^+^ macrophage differentiation was detected 4 days later (**Figure 6A**).

Both live and inactivated JEV induced CD3^+^ macrophage differentiation; however, the inactivated virus exerted less of an effect than the live virus. Moreover, L929 supernatant containing M-CSF combined with IL-2, IL-6, or IFN-γ effectively induced CD3^+^ macrophage differentiation. Interestingly, IL-2 or IL-7 combined with M-CSF also induced CD3^+^CD11b^-^ cell generation (**Figures 6B, C**). Thus, the combination of the cytokines M-CSF, IL-2, IL-6, and IFN-γ promoted CD3^+^ macrophage differentiation. Subsequently, RNAs were extracted from JE brain tissue and subjected to real-time PCR. JEV infection significantly upregulated M-CSF, IL-6 and IFN-γ expression in the mouse brain (**Figure 6D**). Previous reports have shown that prolactin, which regulates T cell development and function (50, 51), was upregulated during brain injury (52, 53). However, the real-time PCR results showed a downregulation of prolactin in the JE brain (**Figure 6D**). The results described above suggest that JEV infection increases M-CSF, IL-6 and IFN-γ expression in the brain, which facilitates CD3^+^ macrophage differentiation.

### JEV Induces CCL2/N-CCL2 Production in the Brain

**Figure 7:**
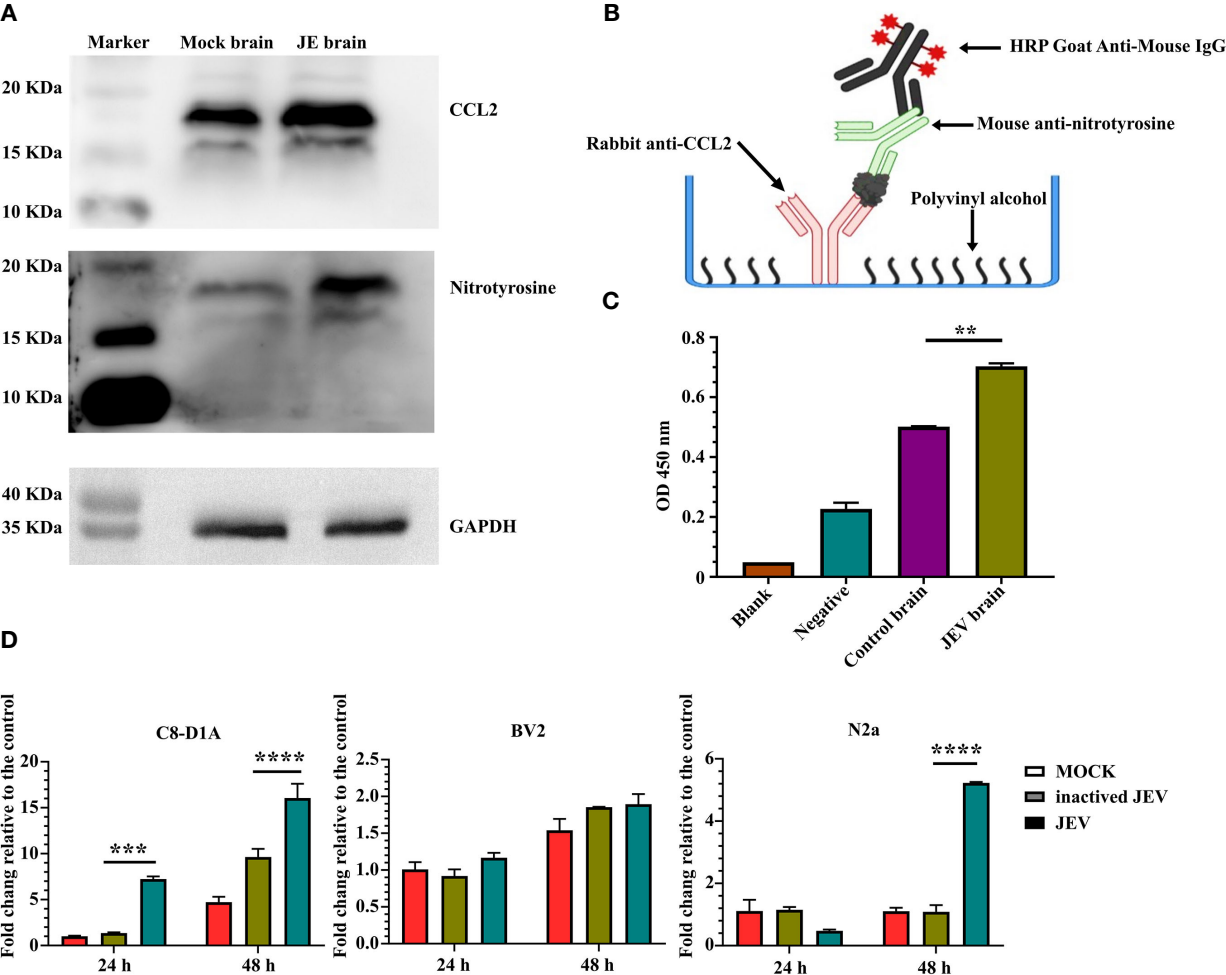
JEV infection in the brain induces CCL2/N-CCL2 production. **(A)** Western blots showing more intense bands for CCL2 and nitrotyrosine at the same location in JE brain samples. **(B, C)** Sandwich ELISA detection of N-CCL2 levels in brain samples. **(B)** Schematic illustration of the N-CCL2 sandwich ELISA. **(C)** Sandwich ELISA detection shows that the JE brain produces more N-CCL2. Blank: No sample or detection antibody added. Negative: No sample added (n = 3). **(D)** Real-time PCR detection of CCL2 expression in astrocyte (C8-D1A), microglia (BV2), and neuronal (N2a) cell lines after JEV infection (MOI = 5) at 24 and 48 hours post-infection (n = 4). Representative results of repeated experiments are shown. **p < 0.01, ***p < 0.001, and ****p < 0.0001.

Flow cytometry analysis confirmed CCR2 expression in M-MDSCs isolated from the JE brain, consistent with previous studies (7, 8). Western blotting also indicated increased protein levels of the CCR2 ligand CCL2 in the JE brain. At the same time, a higher signal for nitrotyrosine was detected with an anti-nitrotyrosine antibody in the JE brain at the site of the CCL2 band (**Figure 7A**), which indicated the presence of N-CCL2 in the JE brain. However, due to the lack of commercial antibodies or detection kits targeting N-CCL2, a sandwich ELISA was developed with rabbit anti-CCL2 and mouse anti-nitrotyrosine antibodies to detect N-CCL2 (**Figure 7B**). The results showed a significant increase in N-CCL2 levels in the JEV-infected mouse brain (**Figure 7C**), which facilitates the infiltration of monocytes rather than T cells (54). Our published report also confirmed that N-CCL2 and CCL2 have the same biological activity as CCR2^+^ cells (55).

Astrocyte (C8-D1A), microglia (BV2), and neuronal cell lines (N2a) were used to detect CCL2 expression and determine its origins during JEV infection. Compared with mock or inactivated virus treatment, live virus induced earlier and higher CCL2 expression in C8-D1A cells. N2a cells also upregulated CCL2 expression in response to virus infection, but the expression was lower and later than that in C8-D1A cells. CCL2 expression in BV2 cells was not changed (**Figure 7D**). Based on these results, after invasion into the brain, JEV induced CCL2 expression in astrocytes. CCL2 was also modified by nitrotyrosine.

## Discussion

Similar to many other viruses, viral immune escape and immune suppression are prerequisites for JEV pathogenicity. JEV employs multiple strategies to evade the innate immune system and block the interferon pathway to escape host immune surveillance (56, 57). However, far fewer studies have examined the mechanism by which JEV regulates cellular immune evasion and the transition between peripheral immunosuppression and CNS immune cell activation. As shown in the present study, JEV hijacks host immune cell development and differentiation to enhance its pathogenic potency. Our data identified M-MDSCs as the major subset of invading cells, which subsequently differentiated into CD3^+^ macrophages in the CNS.

Since the term MDSCs was proposed to describe one heterogeneous group of immature myeloid cells under pathological conditions, MDSCs have become one of the most discussed biological entities in immunology (58). The immunosuppressive capacity of MDSCs has been proven to facilitate angiogenesis, immune evasion of tumor cells and persistence of different viruses. However, in some studies parasitic infection and pneumonitis, MDSC expansion was considered beneficial for the host by promoting the restoration of tissue homeostasis (59–61). Many theories have also been proposed to explain MDSC generation, including cytokines such as GM-CSF, G-CSF, M-CSF, IL-6, PGE-2, IL-10, IL-1β, TGF-β, SCF, VEGF and transcription factors such as STAT3, C/EBPα, C/EBPβ, IRF1, IRF4, IRF8, and mTORC1 (26, 27, 41, 62–69). The heterogeneity of MDSCs has prompted diverse theories of MDSC generation, leading to a lack of general MDSC genetic deletion models. Hence, MDSC-associated studies tend to be limited to specific disease models and conditions. However, the key factor that determines whether immature myeloid cells exhibit suppressive activity remains unknown. Some scholars have proposed that energy metabolism manipulates the fate and function of MDSCs (70, 71), yet this field has not been deeply explored and recognized. In the present study, IRF7 played an important role in JEV-induced M-MDSC generation. In addition, the ZBP1-IRF7 pathway was activated by JEV RNA. However, the M-MDSC RNA-seq results indicated that, in parallel to the upregulation of IRF7, JEV infection did not induce the upregulation of other IRF and interferon genes. Thus, JEV employs multiple strategies to ensure M-MDSC generation. Viral RNA activates IRF7 expression, and other viral factors inhibit interferon and IRF family expression (57, 72, 73).

The M-MDSCs isolated from the JE brain express CCR2. The CCR2 ligand CCL2 was also upregulated in the JE brain. During the inflammatory process, NO production under conditions of oxidative stress may lead to the generation of peroxynitrite and protein nitration (74). Western blotting and ELISAs confirmed the existence of N-CCL2 in JE brain tissues. N-CCL2 has been reported to attract CCR2^+^ myeloid cells but not CCR2^lo^ T lymphocytes (75). The presence of N-CCL2 and M-MDSCs may explain why few classical T cells were detected in the JE mouse brain.

Previous studies have reported the infiltration of Ly-6C^hi^ monocytes, CCR2^+^CD8^+^ T cells, and macrophages in the JE brain (7, 8, 76, 77). The cell types varied in different reports, potentially due to distinct analysis methods and patterns of cell markers. In this study, we identified CNS-infiltrated CD11b^+^Ly-6C^hi^ cells expressing CD3 and F4/80, which usually represent T cells and macrophages, respectively. Although great strides have been made in the field of macrophages, many questions remain unanswered, including the properties of different subpopulations. The common classification of macrophages is based on T helper cells (78). However, M1 and M2 divisions oversimplify the pattern of macrophage populations. Macrophages are also heterogenic phagocytic cells with functional plasticity, which is driven by the environment (79–81). The CD3-TCR complex is necessary for antigen-specific recognition and activation of T cells and has been widely accepted to be uniquely expressed on T cells. However, a small number of studies have shown that CD3-TCR is also expressed on other leukocytes, including neutrophils, eosinophils and macrophages, in human and mouse (82–84). A subsequent study found that human circulating monocytes differentiate into CD3^+^TCRαβ^+^ and CD3^+^TCRαβ^−^ macrophages (20). Thus, we prefer the use of CD3^+^ macrophages rather than TCR^+^ macrophages. In this study, M-CSF, IL-6 and IFN-γ induced CD3^+^ macrophage differentiation in the JE brain microenvironment. The detection of p-SLP76 also suggested that CD3^+^ macrophages exhibited functional activation of TCR signaling. Further studies are required to determine whether other factors regulate CD3^+^ macrophage differentiation.

Numerous studies have revealed the existence of MDSCs in a wide variety of disorders. The regulation of MDSC generation and function remains an area of active study in immunology. At present, only limited knowledge is available on CD3^+^ macrophages. The names of CD3^+^ macrophages and TCR^+^ macrophages are currently interchanged. However, evidence indicates CD3 and TCR expression on nonlymphoid cells. This study focused on M-MDSC generation, migration and CD3^+^ macrophage differentiation in a mouse model of JEV infection, further illustrating the mechanism employed by JEV to evade immune surveillance and deepening the understanding of neuroinflammation in CNS. These findings are expected to provide support for potential adjunct therapy for JE and provide insights into other viral diseases.

## Data Availability Statement

The datasets presented in this study can be found in online repositories. The names of the repository/repositories and accession number(s) can be found in the article/**Supplementary Material**.

## Ethics Statement

All animal experiments were approved by the Research Ethics Committee, Huazhong Agricultural University, Hubei, China (HZAUMO-2019-060) and were performed in accordance with the Guidelines for the Care and Use of Laboratory Animals of the Research Ethics Committee, Huazhong Agricultural University, Hubei, China.

## Author Contributions

MC conceived and guided the study. NZ completed the main part of this work. XG and WZ performed the animal experiments, including establishing the splenectomy model and collecting samples. JX performed the recombinant virus rescue experiments and JEV-PrM IF assay. XC performed the RNA-seq data analysis and uploading. All authors contributed to the article and approved the submitted version.

## Conflict of Interest

The authors declare that the research was conducted in the absence of any commercial or financial relationships that could be construed as a potential conflict of interest.

## Publisher’s Note

All claims expressed in this article are solely those of the authors and do not necessarily represent those of their affiliated organizations, or those of the publisher, the editors and the reviewers. Any product that may be evaluated in this article, or claim that may be made by its manufacturer, is not guaranteed or endorsed by the publisher.

## Supplementary Material

Supplementary Figure 1 Establishment of a mouse GFP-positive bone marrow transplantation model. **(A)** Flow cytometry detection of GFP-positive cells in the peripheral blood of bone marrow-transplanted mice. **(B)** Bright field image and **(C)** GFP fluorescence image of isolated cells infiltrating the brains of JE mice under fluorescence microscopy.

Supplementary Figure 2 Flow cytometry sorting strategy for CD8^+^ T cells and M-MDSCs. **(A)** Flow cytometry sorting strategy for CD8^+^ T cells from the spleen. **(B, C)** Flow cytometry sorting strategy for M-MDSCs from spleen **(B)** and brain **(C)**.

Supplementary Figure 3 JEV-induced MDSCs mainly originate from bone marrow. **(A, B)** Normal and splenectomized mice were infected with 5.0 × 10^4^ PFU JEV per mouse *via* the tail vein. **(A)** Survival curves, percentage of initial body weight, and changes in clinical scores were used to evaluate the effect of splenectomy on JE progression. Scoring standard: 0.5, paw flutters, head shakes; 1, lower activity levels than mock-infected mice; 2, arched back, slow movement; 3, shivering, limpness, paralysis; 4, recumbency, moribund; and 5, death (n = 15). **(B)** Effect of splenectomy on the distribution of MDSCs in the blood, spleen, and bone marrow.

Supplementary Figure 4 Rescue and identification of different rAT mutant viruses. **(A)** PCR detection of NS1'^Δ^ and NS5^ex^ mutations in different infectious plasmid clones. **(B)** Double digestion identification of different infectious plasmid clones with BamHI and MluI. **(C)** Indirect immunofluorescence staining for the PrM protein expressed by different rAT mutant viruses in BHK-21 cells. **(D)** Sequencing identification of NS2A and NS5 segments in different rAT mutant viruses.

Supplementary Figure 5 JEV upregulates the ZBP1-IRF7 signaling pathway in MDSCs. Cluster heat map showing the mRNA expression of the IRF family **(A)** and interferon genes **(B)** between JEV-P3 induced MDSCs and other samples; other IRF genes and interferon genes were not upregulated in P3 MDSCs. GSEA **(C)** and projection **(D)** indicate that IRF7, including the cytosolic DNA-sensing pathway (KEGG map04623), was upregulated in JEV-P3 induced MDSCs.

Supplementary Figure 6 IRF7-V1 isoform overexpression plays an important role in M-MDSC generation and function. **(A)** Flow cytometry gating strategy for ex vivo lentivirus induction of MDSCs. **(B)** Representative scatter plots and **(C)** bar chart showing that ZBP1 and IRF7 overexpression increased the M-MDSC proportion in the ex vivo induction system at 4 days post-infection (n = 4). **(D)** Real-time PCR analysis showing that IRF7-V1 overexpression upregulates ARG1 and iNOS at 4 days post-infection (n = 4). Representative results of repeated experiments are shown.

Supplementary Figure 7 CD3^+^F4/80^+^ cells exhibit higher proliferative and phagocytic capacities. **(A, B)** Flow cytometry analysis of CFSE-labeled macrophages after stimulation with 2.5 μg/mL ConA for 48 hours. **(A)** Representative histograms and **(B)** bar chart showing that JBS-induced macrophages have a higher proliferative capacity and response to ConA stimulation (n = 4). **(C)** The bar chart shows that JBS-induced CD3^+^ macrophages have a higher fluorescence microsphere phagocytic capacity (n = 4).

Supplementary Figure 8 The ZBP1-IRF7 signaling pathway is upregulated in CD3^+^F4/80^+^ cells. GSEA **(A)** and projection **(B)** show that IRF7, including the cytosolic DNA-sensing pathway (KEGG map04623), was upregulated in CD3^+^F4/80^+^ cells.
