# Supplementary figures and images for: JEV Infection Induces M-MDSC Differentiation Into CD3+ Macrophages in the Brain
Source: Front Immunol. 2022 Apr 21;13:838990. doi: 10.3389/fimmu.2022.838990 (PMC9068957; doi:10.3389/fimmu.2022.838990)

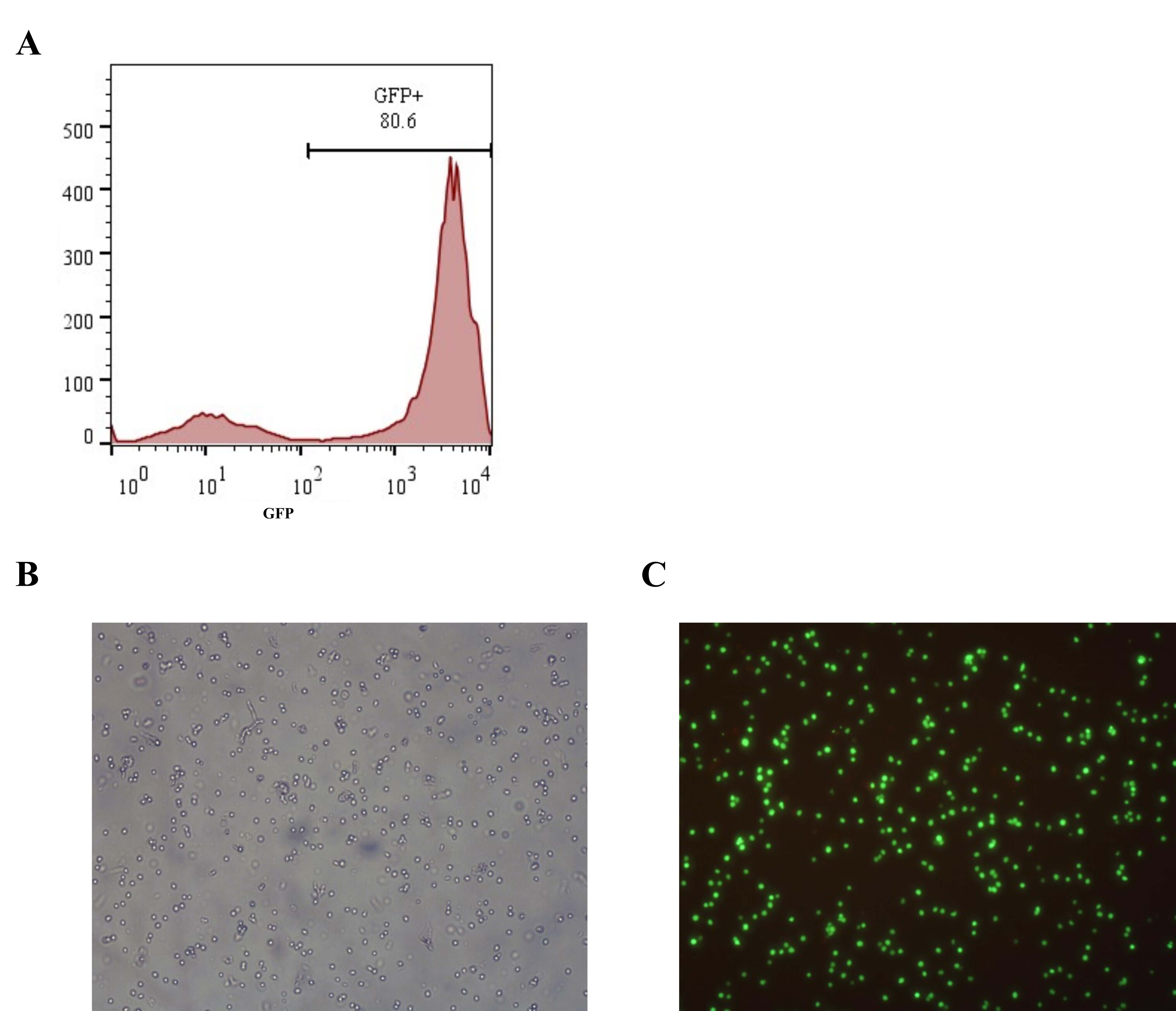

Supplement: Supplementary Figure 1 — Establishment of a mouse GFP-positive bone marrow transplantation model. (A) Flow cytometry detection of GFP-positive cells in the peripheral blood of bone marrow-transplanted mice. (B) Bright field image and (C) GFP fluorescence image of isolated cells infiltrating the brains of JE mice under fluorescence microscopy. [file Image_1.jpeg]

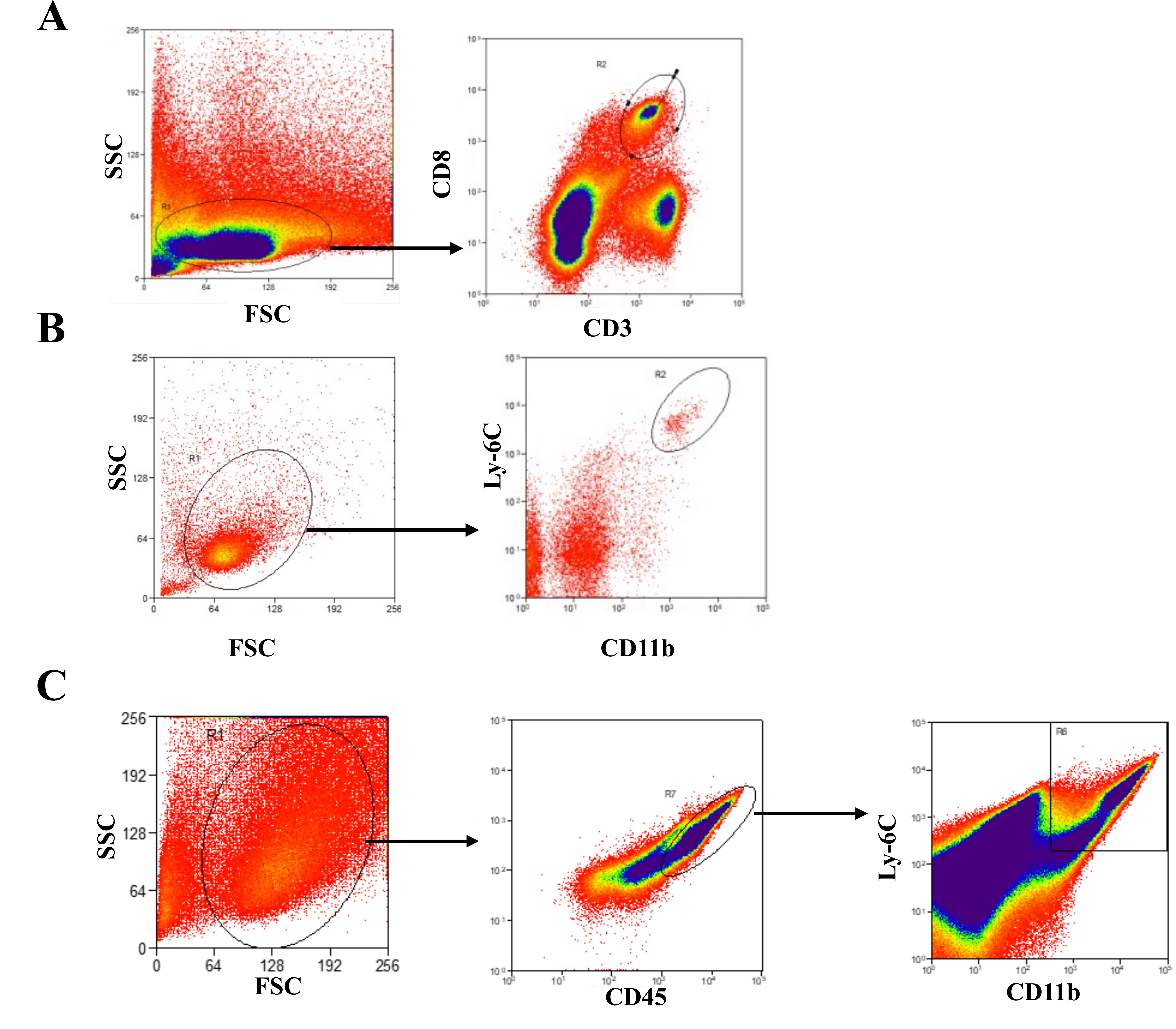

Supplement: Supplementary Figure 2 — Flow cytometry sorting strategy for CD8+ T cells and M-MDSCs. (A) Flow cytometry sorting strategy for CD8+ T cells from the spleen. (B, C) Flow cytometry sorting strategy for M-MDSCs from spleen (B) and brain (C). [file Image_2.jpeg]

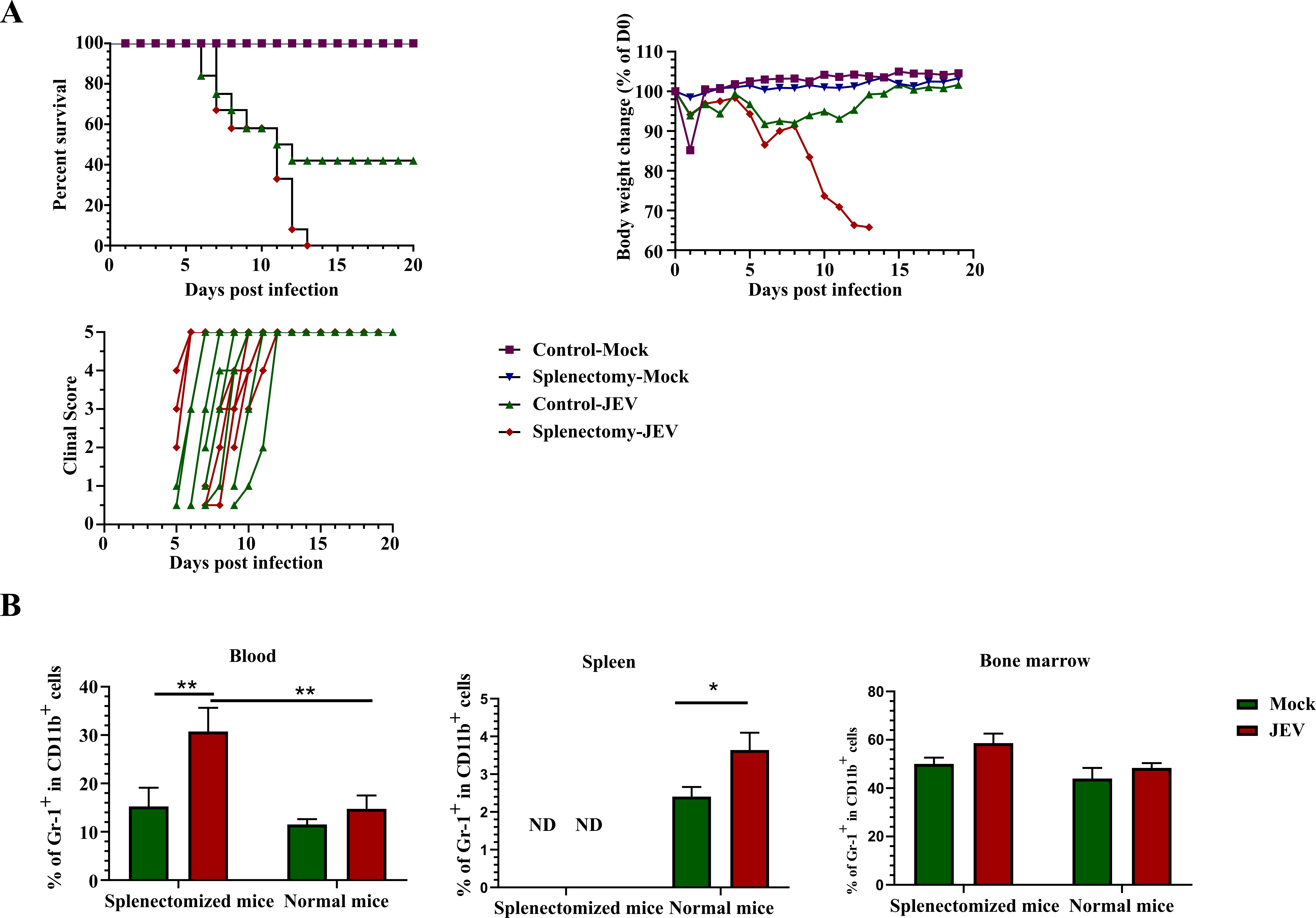

Supplement: Supplementary Figure 3 — JEV-induced MDSCs mainly originate from bone marrow. (A, B) Normal and splenectomized mice were infected with 5.0 × 10^4^ PFU JEV per mouse via the tail vein. (A) Survival curves, percentage of initial body weight, and changes in clinical scores were used to evaluate the effect of splenectomy on JE progression. Scoring standard: 0.5, paw flutters, head shakes; 1, lower activity levels than mock-infected mice; 2, arched back, slow movement; 3, shivering, limpness, paralysis; 4, recumbency, moribund; and 5, death (n = 15). (B) Effect of splenectomy on the distribution of MDSCs in the blood, spleen, and bone marrow. [file Image_3.jpeg]

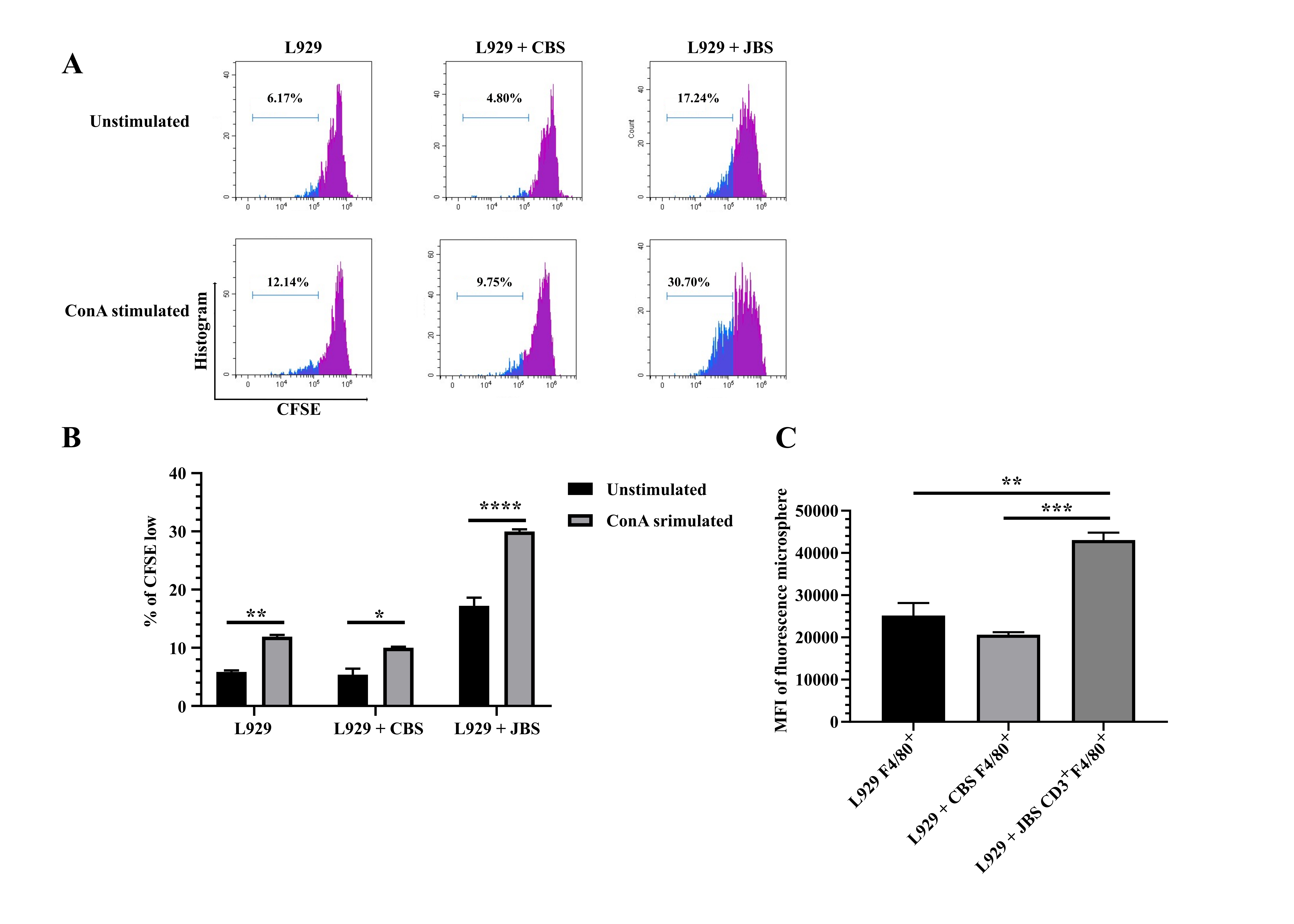

Supplement: Supplementary Figure 7 — CD3+F4/80+ cells exhibit higher proliferative and phagocytic capacities. (A, B) Flow cytometry analysis of CFSE-labeled macrophages after stimulation with 2.5 μg/mL ConA for 48 hours. (A) Representative histograms and (B) bar chart showing that JBS-induced macrophages have a higher proliferative capacity and response to ConA stimulation (n = 4). (C) The bar chart shows that JBS-induced CD3+ macrophages have a higher fluorescence microsphere phagocytic capacity (n = 4). [file Image_7.jpeg]

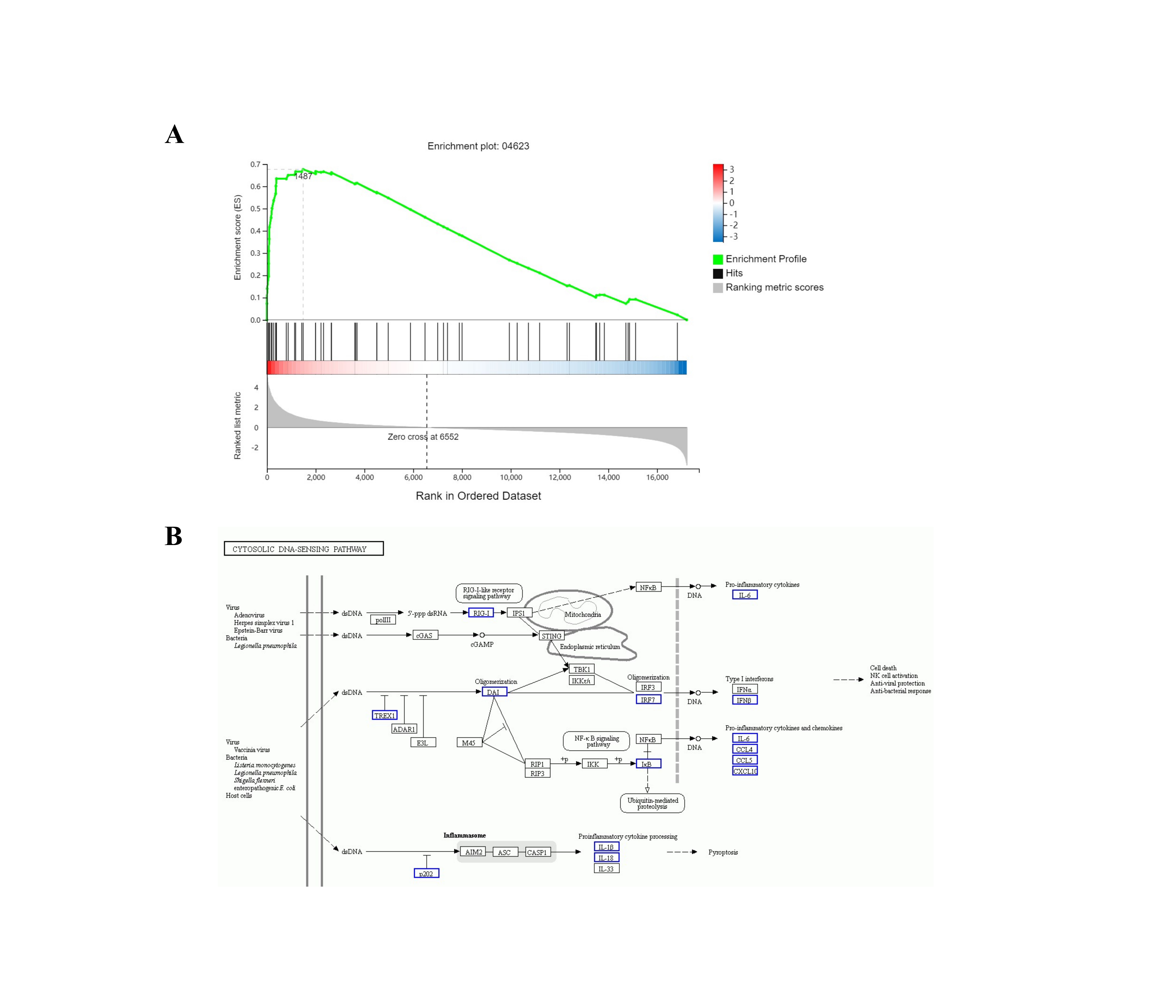

Supplement: Supplementary Figure 8 — The ZBP1-IRF7 signaling pathway is upregulated in CD3+F4/80+ cells. GSEA (A) and projection (B) show that IRF7, including the cytosolic DNA-sensing pathway (KEGG map04623), was upregulated in CD3+F4/80+ cells. [file Image_8.jpeg]
