# Supplementary material for: JEV Infection Induces M-MDSC Differentiation Into CD3+ Macrophages in the Brain
Source: Front Immunol. 2022 Apr 21;13:838990. doi: 10.3389/fimmu.2022.838990 (PMC9068957; doi:10.3389/fimmu.2022.838990)
Supplement: Supplementary file 9 [file DataSheet_1.docx]

**Supplemental Table 1. Antibodies used for Western blotting and ELISAs**

| **Antibodies** | **Source** | **Identifier** |
| --- | --- | --- |
| Rabbit monoclonal anti-IRF7 | Zen bioscience | Cat# R26145; RRID: AB_2892077 |
| Rabbit monoclonal anti-ZAP70, Clone D1C10E | Cell Signaling Technology | Cat# 3165; RRID: AB_2218656 |
| Rabbit polyclonal anti-P-SLP76 (Tyr145) | Cell Signaling Technology | Cat# 14770; RRID: AB_2798604 |
| Rabbit polyclonal anti-CCL2 | Proteintech | Cat# 25542-1-AP; RRID: AB_2880126 |
| Goat polyclonal anti-CCL2 | R&D Systems | Cat# AF-479-NA; RRID: AB_354500 |
| Rabbit polyclonal anti-nitrotyrosine | Sigma-Aldrich | Cat# N0409; RRID: AB_260745 |
| Mouse monoclonal anti-nitrotyrosine, Clone 39B6 | SANTA CRUZ | Cat# sc-32757; RRID: RRID: AB_628022 |

**Supplemental Table 2. Antibodies used for flow cytometry**

| **Antibodies** | **Source** | **Identifier** |
| --- | --- | --- |
| Anti-B220-PE-Vio 770, Clone REA755 | Miltenyi Biotec | Cat# 130-110-711; RRID: AB_2658285 |
| Anti-CD3-PE-Vio 770, Clone 145-2C11 | Miltenyi Biotec | Cat# 130-102-794; RRID: AB_2660399 |
| Anti-CD3-APC, Clone 17A2 | BioLegend | Cat# 100236; RRID: AB_2561456 |
| Anti-CCR2-PE, Clone 475301 | R&D Systems | Cat# FAB5538P; RRID: AB_10718414 |
| Anti-CD4-APC-H7, Clone GK1.5 | BD Biosciences | Cat# 560181; RRID: AB_1645235 |
| Anti-CD8a-Pacific Blue, Clone 53-6.7 | BioLegend | Cat# 100725; RRID: AB_493425 |
| Anti-CD200-APC, Clone OX-90 | BioLegend | Cat# 123809; RRID: AB_10900996 |
| Anti-CD335-PerCP-Cy5.5, Clone 29A1.4 | BioLegend | Cat# 137610; RRID: AB_10641137 |
| Anti-F4/80-PE, Clone BM8 | BioLegend | Cat# 123109; RRID: AB_893498 |
| Anti-F4/80-PE-eFluor 610, Clone BM8 | eBioscience | Cat# 61-4801; RRID: AB_2574611 |
| Anti-F4/80-Alexa Fluor 647, Clone BM8 | Invitrogen | Cat# MF48021; RRID: AB_10375289 |
| Anti-F4/80-Brilliant Violet 421, Clone BM8 | BioLegend | Cat# 123131; RRID: AB_10901171 |
| Anti-NK1.1-PE, Clone PK136 | eBioscience | Cat# 12-5941; RRID: RRID: AB_466050 |
| Anti-CD11b-PE, Clone M1/70 | BioLegend | Cat# 101207; RRID: AB_312790 |
| Anti-CD11b-PerCP-Cy5.5, Clone M1/70 | BioLegend | Cat# 101228 ; RRID: AB_893232 |
| Anti-Gr-1-APC, Clone RB6-8C5 | BioLegend | Cat# 108412; RRID: AB_313377 |
| Anti-Ly-6C-PE, Clone, HK1.4 | BioLegend | Cat# 128008; RRID: RRID: AB_1186132 |
| Anti-Ly-6C-APC, Clone, HK1.4 | BioLegend | Cat# 128016; RRID: AB_1732076 |
| Anti-Ly-6G-Pacific Blue, Clone, 1A8 | BioLegend | Cat# 127612; RRID: RRID: AB_2251161 |
| Anti-TCR β-APC, Clone, H57-597 | BioLegend | Cat# 109212; RRID: RRID: AB_313435 |
| Anti-TCRγ/δ-PerCP/Cy5.5, Clone, GL3 | BioLegend | Cat# 118118; RRID: AB_10612756 |

**Supplemental Table 3. BioSample accession numbers used in this article**

| **Sample name** | **Accession number** |
| --- | --- |
| P3 MDSCs | SAMN19795974 |
| CD45 | SAMN19795975 |
| control MDSCs | SAMN19795976 |
| HSC | SAMN19795977 |
| cKit Sca1 Lin- | SAMN19795978 |
| L929 F4/80 | SAMN19803034 |
| L929 + JBS CD3F4/80 | SAMN19803035 |

**Supplemental Table 4. Real-time PCR primers**

| **Target** | **Forward sequence (5′-3′)** | **Reverse sequence (5′-3′)** |
| --- | --- | --- |
| β-actin | CATCCGTAAAGACCTCTATGCCAAC | ATGGAGCCACCGATCCACA |
| RPL7L1 | ACGGTGGAGCCTTATGTGAC | TCCGTCAGAGGGACTGTCTT |
| ARG1 | CCAGAAGAATGGAAGAGTCAGTGT | GCAGATATGCAGGGAGTCACC |
| iNOS | GGCAGCCTGTGAGACCTTTG | TTGCATTGGAAGTGAAGCGTT |
| ZBP1 | GAAATGCCAAGTGCCCAAGAA | CCCGCCTATGCTCCATGTT |
| IRF7 | CGACTGCCTGCGTGATGCTA | CTTGTCCTGGCTGATGAGATAGTGGTA |
| PHF11b | TCATCAGGACTGGTGGAGAG | AACTTGTAGAATTGCGTGGTCTT |
| CD3e | GGTGCTCCAGGATTTCTCGG | GCCTTGGCCTTCCTATTCTTG |
| ZAP70 | CGACTGCCTGCGTGATGCTA | CTTGTCCTGGCTGATGAGATAGTGGTA |
| M-CSF | GTGTCAGAACACTGTAGCCAC | TCAAAGGCAATCTGGCATGAAG |
| IL-2 | GTGCTCCTTGTCAACAGCG | GGGGAGTTTCAGGTTCCTGTA |
| IL-6 | TAGTCCTTCCTACCCCAATTTCC | TTGGTCCTTAGCCACTCCTTC |
| IFN-γ | GCCACGGCACAGTCATTGA | TGCTGATGGCCTGATTGTCTT |
| Prolactin | CCAATCTGTTCCGCTGGTGACT | GGCTTGTTCCTTGTCTTCAGGTGTA |
